# MicroRNA-9 expression is a prognostic biomarker in patients with osteosarcoma

**DOI:** 10.1186/1477-7819-12-195

**Published:** 2014-06-27

**Authors:** Shi-hong Xu, Yong-liang Yang, Shu-mei Han, Zong-hui Wu

**Affiliations:** 1 Department of Orthopedics, Shandong Provincial Hospital affiliated to Shandong University, Jinan, Shandong 250021, China; 2 Department of Medicine, Shandong Cancer Hospital and Institute, Jinan, Shandong 250117, China; 3 Department of ultrasound diagnosis, Shandong Medical Imaging Research Institute, Shandong University, 324 Jingwu Road, Jinan, Shandong Province 250021, China

**Keywords:** Osteosarcoma, MicroRNA-9, Prognosis

## Abstract

**Background:**

The purpose of the present study was to examine the expression levels of microRNA-9 (miR-9) in osteosarcoma tissues and normal bone tissues, and investigate the relationships between miR-9 expression, clinicopathological features and the prognosis of patients with osteosarcoma.

**Methods:**

The expression levels of miR-9 in osteosarcoma tissues and corresponding non-cancerous tissues were detected using a real-time quantitative assay. Differences in patient survival were determined using the Kaplan–Meier method and a log-rank test. A Cox proportional hazards regression analysis was used for univariate and multivariate analyses of prognostic values.

**Results:**

Compared to non-cancerous bone tissues, the expression levels of miR-9 in osteosarcoma tissues were significantly elevated (*P* < 0.001). We found that the expression level of miR-9 was significantly associated with tumor size (*P* = 0.011), clinical stage (*P* = 0.009) and distant metastasis (*P* < 0.001). The Kaplan–Meier curve showed that patients with low miR-9 expression survived significantly longer than patients with high miR-9 expression (*P* = 0.0017). Multivariate analysis suggested that miR-9 expression level (*P* = 0.002) is an independent prognostic factors for overall survival.

**Conclusions:**

The findings of our study suggest that increased miR-9 expression has a strong correlation with the aggressive progression of osteosarcoma and its overexpression is a statistically significant risk factor affecting overall survival, suggesting that increased miR-9 expression could be a valuable marker of tumor progression and for prognosis of osteosarcoma.

## Background

Osteosarcoma is the most common primary malignancy in children and adolescents, accounting for 20 to 35% of all malignant primary bone tumors [1-3]. Although considerable advances in tumor excision technology, adjuvant chemotherapy and radiotherapy have significantly increased the survival rate of patients with osteosarcoma, the survival of osteosarcoma patients with lung metastasis and at an advanced clinical stage is quite poor [4]. A greater understanding of osteosarcoma is essential for developing novel approaches to increase survival rates. To our disappointment, despite the various efforts of basic research and clinical practice, the molecular genetic mechanisms and the biology involved in osteosarcoma remain poorly understood [5].

MicroRNAs (miRNAs) are small non-coding RNA molecules that play an important role in the regulation of mRNA expression. miRNAs are known to be involved in various cellular processes and are associated with various diseases including cancer [6-8]. The association of altered microRNA expression with cancerogenesis as well as tumor progression is well established [9-11]. A growing number of microRNAs have been classified as oncogenes or tumor-suppressor genes. In addition, miRNA expression profiles and specific miRNAs have been shown to be potential diagnostic or prognostic tools for cancer [12-14].

Previous studies have found that miR-9 is downregulated in several cancers, including ovarian cancer, colon cancer, gastric cancer, renal cancer and esophageal cancer [15-19]. However, the expression of miR-9 has been found to be upregulated in biliary tract cancer, breast cancer, brain tumor and lung cancer [20-24]. These results suggest that miR-9 may play pivotal roles in tumorigenesis and tumor progression, and also exert different effects in various types of cancer. Hu *et al*. found that the expression level of miR-9 was increased in an osteosarcoma cell line compared with an osteoblast cell line [25]. However, the clinical significance of miR-9 in human osteosarcoma has not been investigated deeply. In the present study, we examined the expression levels of miR-9 in osteosarcoma tissues and normal bone tissues, and investigated the relationships between miR-9 expression, clinicopathological features and the prognosis of patients with osteosarcoma.

## Methods

### Patients and tissue samples

This study was approved by the Research Ethics Committee of Shandong Provincial Hospital affiliated to Shandong University. Written informed consent was obtained from all of the patients. All specimens were handled and made anonymous according to the ethical and legal standards. For quantitative real-time reverse-transcriptase-polymerase chain reaction (qRT-PCR) analysis, 79 patients with osteosarcomas and corresponding non-cancerous bone tissue samples from the same specimens were collected at Shandong Provincial Hospital affiliated to Shandong University from June 2006 to July 2012. No patients had received radiotherapy or chemotherapy before surgery. The clinical stage of these osteosarcoma patients was classified according to the sixth edition of the tumor-node-metastases classification of the Union for International Cancer Control. The clinicopathological information of the patients is shown in Table 1.

**Table 1:** Correlation of miR-9 expression levels with clinicopathological features of patients with osteosarcoma

**Clinicopathological features**	**Number of cases**	**miR-9 expression**		***P *** **value**
		**High ( *n *, %)**	**Low ( *n *, %)**	
Age				
<50	41	23 (56.1%)	18 (43.9%)	0.879
≥50	38	17 (44.7%)	21 (55.3%)	
Gender				
Male	44	25 (56.8%)	19 (43.2%)	0.636
Female	35	15 (42.9%)	20 (57.1%)	
Anatomical location				
Tibia/femur	51	26 (51.0%)	25 (49.0%)	0.901
Elsewhere	28	14 (50.0%)	14 (50.0%)	
Tumor size (cm)				
<8	48	17 (35.4%)	31 (64.6%)	
≥8	31	23 (74.2%)	8 (25.8%)	0.011
Clinical stage				
I/II	39	9 (23.1%)	30 (76.9%)	
III	40	31 (77.5%)	9 (22.5%)	0.009
Pathological fracture				
Present	11	6 (54.5%)	5 (45.4%)	
Absent	68	34 (50.0%)	34 (50.0%)	0.899
Distant metastasis				
Present	19	17 (89.5%)	2 (10.5%)	
Absent	60	21 (35.0%)	39 (65.0%)	<0.001

### miRNA qRT-PCR assay

The expression levels of miR-9 in osteosarcoma and corresponding non-cancerous tissues were detected using a qRT-PCR assay. Briefly, total RNA from tissue samples was extracted with TRizol reagent (Invitrogen, Breda, the Netherlands) according to the manufacturer’s instructions. cDNA was reverse transcribed from total RNA samples using specific miRNA primers from the TaqMan MicroRNA Assays and reagents from the TaqMan MicroRNA Reverse Transcription kit (Applied Biosystems, Foster City, CA, USA) according to the manufacturer’s instructions. Products were amplified by PCR using TaqMan Universal PCR Master Mix kit (Applied Biosystems). The quantitative PCR was performed with the specific primers as follows: miR-9_F, 5′-GTGCAGGGTCCGAGGT; miR-9_R, 5′-GCGCTCTTTGGTTATCTAGC-3′; U6_F, 5′-CTCGCTTCGGCAGCACA-3′; U6_R, 5′-AACGCTTCACGAATTTGCGT-3′. Small nucleolar RNA U6 was used as an internal standard for normalization. The cycle threshold (*C*_*T*_) was calculated. The 2^-ΔCT^ (Δ*C*_*T*_ = *C*_*T*miR-9_ – *C*_*T*U6 RNA_) method was used to quantify the relative amount of miR-9. In addition, each measurement was performed in triplicate.

### Statistical analysis

SPSS 13.0 statistical software (SPSS Inc, Chicago, IL, USA) was used for the statistical assay of all experimental data. Continuous variables were expressed as mean ± standard deviation (SD). The paired *t*-test was used to evaluate the differences in miR-9 expression levels in osteosarcoma and corresponding non-cancerous bone tissues. The chi-square test was used to show differences in categorical variables. Differences in patient survival were determined by the Kaplan–Meier method and log-rank test. A Cox proportional hazards regression analysis was used for univariate and multivariate analyses of prognostic values. A difference was considered statistically significant when *P* < 0.05.

## Results

### Elevated expression of miR-9 in osteosarcoma tissues

**Figure 1:**
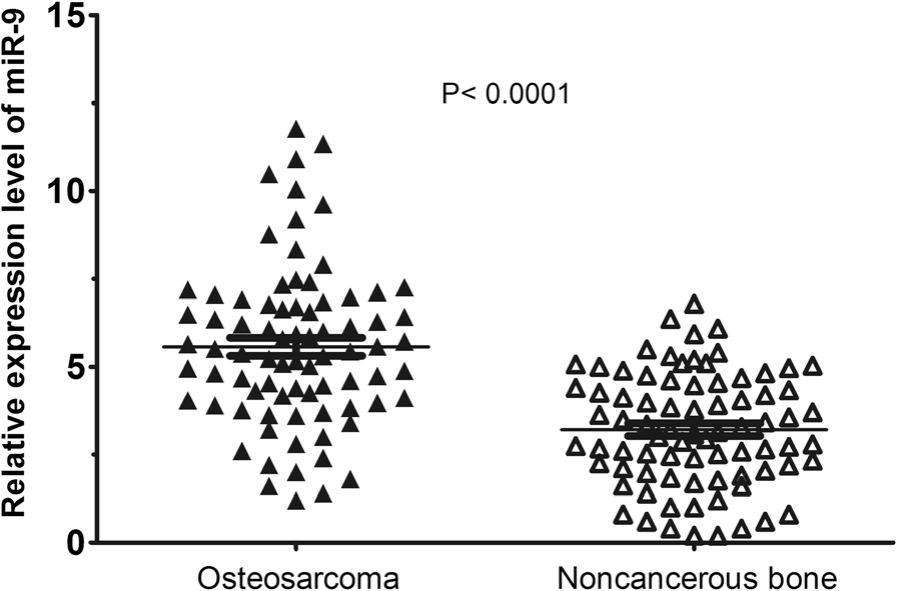
**miR-9 expression in 79 pairs of osteosarcoma tissues and corresponding non-cancerous tissues.** Compared to non-cancerous bone tissues, the expression levels of miR-9 in osteosarcoma tissues were significantly elevated (*P* < 0.001).

The expression levels of miR-9 in osteosarcoma and corresponding non-cancerous bone samples were detected by qRT-PCR and normalized to RNU6. Compared to non-cancerous bone tissues, the expression levels of miR-9 in osteosarcoma tissues were significantly elevated (*P* < 0.001, Figure 1). The relative level of miR-9 expression normalized to RNU6 in osteosarcoma tissues (mean ± SD: 5.57 ± 2.28) was significantly higher than that in corresponding non-cancerous bone tissues (mean ± SD: 3.21 ± 1.61). The median of miR-9 expression levels in all 79 patients with osteosarcoma was 5.44. We divided the patients into two groups according to their expression levels of miR-9 using the median as a cutoff: high miR-9 expression group (*n* = 40, mean ± SD: 7.30 ± 1.69) and low miR-9 expression group (*n* = 39, mean ± SD: 3.80 ± 1.18).

### Correlation between clinicopathological features and miR-9 expression levels in osteosarcoma tissues

To further delineate the possible roles of miR-9 in the development and progression of osteosarcoma, we conducted an investigation into the associations of miR-9 expression with clinicopathological features of the patients with osteosarcoma. Table 1 summarizes the associations of miR-9 expression with various clinicopathological parameters of the patients with osteosarcoma. We found that the expression level of miR-9 was significantly associated with tumor size (*P* = 0.011), clinical stage (*P* = 0.009) and distant metastasis (*P* < 0.001). In contrast, no association was found between the expression level of miR-9 with age (*P* = 0.879), gender (*P* = 0.636), anatomical location (*P* = 0.901) or pathological fracture (*P* = 0.899).

### MiR-9 expression is a prognostic biomarker in patients with osteosarcoma

**Figure 2:**
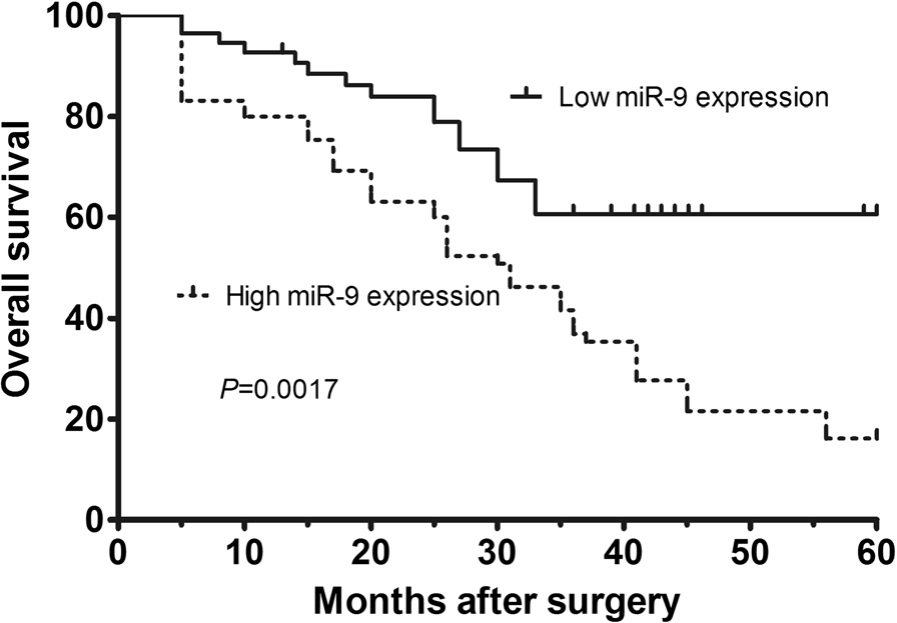
Kaplan–Meier survival curves for osteosarcoma patients with high or low expression of miR-9.

The correlation between miR-9 expression level and survival time of the patients with osteosarcoma was evaluated using Kaplan–Meier survival analysis. The Kaplan–Meier curve for overall survival regarding miR-9 expression level is shown in Figure 2. The curve shows that osteosarcoma patients with low miR-9 expression in their tumor tissues survived significantly longer than patients with high miR-9 expression (*P* = 0.0017, by log-rank test). For patients with high miR-9 expression, the 5-year overall survival was 16.2%; however, the overall survival of patients with low miR-9 expression was 60.6%. Multivariate analysis suggested that clinical stage (*P* = 0.01) and miR-9 expression level (*P* = 0.002) were significant independent prognostic factors for overall survival (shown in Table 2).

**Table 2:** Multivariate analysis of overall survival in patients with osteosarcoma

**Parameter**	**Hazard ratio**	**95% confidence interval**	***P *** **value**
Age	1.06	0.62–2.98	0.67
Gender	0.91	0.67–1.84	0.53
Anatomical location	0.95	0.81–2.01	0.79
Tumor size (cm)	2.56	0.91–5.39	0.08
Clinical stage	3.31	1.93–6.77	0.01
Pathological fracture	1.27	0.59–2.33	0.49
Distant metastasis	4.01	0.98–10.89	0.06
miR-9 expression level	4.77	2.86–5.91	0.002

## Discussion

miRNAs are known to be involved in various cellular processes and are associated with various diseases including cancer. They can regulate gene expression at a post-transcriptional level and play a pivotal role in the regulation of cell development, metabolism, immunity, proliferation, differentiation and apoptosis. Published data shows that miRNAs are involved in carcinogenesis as either oncogenes or tumor suppressors and many cancer-related miRNAs have been identified functionally [26,27].

Many molecular markers have proven prognostic value for osteosarcoma. It would be helpful if these markers were used as objective instruments for predicting the chance of survival or chemotherapy response, especially early in treatment, preferably even before surgery. Previous studies have found that miR-9 is downregulated in several cancers, including ovarian cancer, colon cancer, gastric cancer, renal cancer and esophageal cancer [15-19]. However, the expression of miR-9 has been found to be upregulated in biliary tract cancer, breast cancer, brain tumor and lung cancer [20-24]. These results suggest that miR-9 may play pivotal roles in tumorigenesis and tumor progression, and also exert different effects in various types of cancer. In the present study, we found that miR-9 expression was increased in osteosarcoma tissues compared with non-cancerous bone tissues; further, the upregulation of miR-9 in osteosarcoma tissues was significantly correlated with aggressive clinicopathological features, including tumor size, clinical stage and distant metastasis. These findings suggested that a higher level of miR-9 expression may be involved in osteosarcoma pathogenesis and progression. Furthermore, we analyzed a correlation between miR-9 expression level and prognosis of osteosarcoma. Patients with high miR-9 expression had a shorter overall survival rate than those with low miR-9 expression. These findings were further supported by the multivariate analyses of a Cox proportional hazards regression model, suggesting that the level of miR-9 expression may be an independent factor for predicting the prognosis of patients with osteosarcoma. This was in agreement with previous studies that validated miR-9 as a novel prognostic biomarker for lung cancer, cervical cancer and glioma [20,28,29].

Therefore, our data suggest that the high expression of miR-9 is associated with an increased risk of death from osteosarcoma. To our knowledge, this is the first study to investigate the clinical significance of miR-9 in patients with osteosarcoma. There are several possible causes for the dysregulation of miRNA profiling, including miR-9, in diverse carcinomas. miRNA expression is regulated by genetic and epigenetic factors. For instance, MYC/MYCN regulates the expression of miR-9 in breast cancer, and DNA methylation influences miR-9 expression in colorectal cancer [21,30]. Regarding the possible molecular mechanisms regulated by miR-9 expression in human cancers, Ma *et al*. reported that miR-9 is a putative metastasis promoter in breast cancer. They demonstrated that miR-9 targeted CDH-1 mRNA, which encodes E-cadherin, leading to the scattering and EMT-like conversion of SUM149 cells. Ectopic expression of miR-9 downregulated the E-cadherin level, thus increasing the nuclear translocation of β-catenin, and enhanced its binding with transcription factors TCF/LEF to upregulate the transcription of genes that facilitate cell proliferation and angiogenesis [21]. Zheng *et al*. identified cyclin D1 and Ets1 as new targets of miR-9 in gastric cancer and demonstrated that miR-9 suppressed proliferation, invasion and metastasis [15]. Guo *et al*. reported that miR-9 inhibited ovarian cancer cell growth through regulation of NF-κB1 [18]. In this study, we identified that miR-9 played an oncogenic role in osteosarcoma; however, the precise mechanism of miR-9 in osteosarcoma tumorigenesis and progression is still not understood.

## Conclusions

In conclusion, the findings of our study suggest that increased miR-9 expression has a strong correlation with the aggressive progression of osteosarcoma and its overexpression is a statistically significant risk factor affecting overall survival in patients with osteosarcoma, suggesting that increased miR-9 expression could be a valuable marker of tumor progression and for prognosis of osteosarcoma. Further research is needed to clarify the exact mechanism of miR-9 in osteosarcoma.

## Abbreviations

miRNA: microRNA; miR-9: microRNA-9; qRT-PCR: quantitative real-time reverse-transcription-polymerase chain reaction; SD: standard deviation.

## Competing interests

The authors have no proprietary interest in any materials or methods described within this paper. This submission has not been published anywhere previously and it is not simultaneously being considered for any other publication.

## Authors’ contributions

XS, YY, HS and WZ carried out the experiments, XS designed the study, XS and YY prepared the manuscript. All authors read and approved the final manuscript.
